# Occupational exposure to vapors, gasses, dusts, and fumes in relation to causes of death during 24 years in Helsinki, Finland

**DOI:** 10.1007/s00420-023-02031-1

**Published:** 2023-12-19

**Authors:** Juuso Jalasto, Ritva Luukkonen, Ari Lindqvist, Arnulf Langhammer, Hannu Kankaanranta, Helena Backman, Eva Rönmark, Anssi Sovijärvi, Päivi Piirilä, Paula Kauppi

**Affiliations:** 1 Department of Clinical Physiology, HUS Medical Diagnostic Center, Helsinki University Central Hospital and University of Helsinki, PL 281, 00029 HUS Helsinki, Finland; 2 Finnish Institute of Occupational Health, Helsinki, Finland; 3 Department of Pulmonary Medicine, Heart and Lung Center, Helsinki University Hospital and Helsinki University, Helsinki, Finland; 4 Department of Public Health and Nursing, HUNT Research Centre, NTNU, Norwegian University of Science and Technology, Levanger, Norway; 5 Levanger Hospital, Nord-Trøndelag Hospital Trust, Levanger, Norway; 6 Krefting Research Centre, Institute of Medicine, Department of Internal Medicine and Clinical Nutrition, University of Gothenburg, Gothenburg, Sweden; 7 Department of Respiratory Medicine, Seinäjoki Central Hospital, Seinäjoki, Finland; 8 Respiratory Research Group, Faculty of Medicine and Health Technology, Tampere University, Tampere University, Tampere, Finland; 9 Department of Public Health and Clinical Medicine, Section of Sustainable Health, The OLIN Unit, Umeå University, Umeå, Sweden

**Keywords:** Occupational exposure, Causes of death, Cardiovascular diseases, Respiratory diseases, Dementia diseases

## Abstract

**Purpose:**

Environmental particulate matter (PM) exposure has been shown to cause excess all-cause and disease-specific mortality. Our aim was to compare disease-specific mortality by estimated occupational exposure to vapors, gasses, dusts, and fumes (VGDF).

**Methods:**

The data source is the Helsinki part of the population-based FinEsS study on chronic obstructive pulmonary diseases including information on age, education level, main occupation, sex, and tobacco smoking combined with death registry information. We compared estimated VGDF exposure to mortality using adjusted competing-risks regression for disease-specific survival analysis for a 24-year follow-up.

**Results:**

Compared to the no-exposure group, the high occupational VGDF exposure group had sub-hazard ratios (sHR) of 1.7 (95% CI 1.3–2.2) for all cardiovascular-related and sHR 2.1 (1.5–3.9) for just coronary artery-related mortality. It also had sHR 1.7 (1.0–2.8) for Alzheimer’s or vascular dementia-related mortality and sHR 1.7(1.2–2.4) for all respiratory disease-related mortality.

**Conclusion:**

Long-term occupational exposure to VGDF increased the hazard of mortality- to cardiovascular-, respiratory-, and dementia-related causes. This emphasizes the need for minimizing occupational long-term respiratory exposure to dust, gasses, and fumes.

**Supplementary Information:**

The online version contains supplementary material available at 10.1007/s00420-023-02031-1.

## Introduction

The effect of airborne small particulate matter as a risk for several kinds of diseases has been previously found in studies in relation to air pollution. Especially the effect of exposure to airborne particulate matter in the development of respiratory (Balmes et al. 2003), cardiovascular (Fang et al. 2010), as well as degenerative neurological diseases (Gunnarsson and Bodin 2019; Hayden et al. 2010; Heusinkveld et al. 2016; Killin et al. 2016) has been proposed previously. For occupational exposure, a study from the 1980s already showed association of self-reported occupational dust, gasses, and fumes with coughing, phlegm production, and COPD (Korn et al. 1987).

Alongside self-reported estimates, job-exposure matrixes (JEMs) have been a useful tool for exposure estimation. In this study, we used a JEM based on a study which compared exposure levels to respiratory symptoms and airway health measurements (Sunyer et al. 1998). A similar type of JEM estimation was also used in other studies studying the effect of exposure on chronic lower airway diseases (Matheson et al. 2005; Mehta et al. 2012). These types of JEMs categorize exposure to vapors, gasses, dusts, and/or fumes (VGDF) are considered especially useful in finding exposure related to chronic obstructive respiratory diseases. The usefulness in large studies can be seen in the appliance of the estimation via known and often used occupation classifications such as the International Categorization of Occupations ver. 1988 (ISCO-88) which we have already used in a previous article (Jalasto et al. 2023). In our previous papers, we also estimated the all-cause mortality related to exposure and self-reported respiratory diagnosis, and the result showed increased all-cause mortality related to high exposure to VGDF (Jalasto et al. 2023). In raw numbers, the amount of work-related all-cause mortality in Finland in the 1990s was estimated to be around 1800 per year (Nurminen and Karjalainen 2001). Similarly, the mortality attributed to specific diseases (Respiratory, Cancer and Myocardial infarction) in Sweden has previously been approximated to be around 800 per year (Jarvholm et al. 2013).

A previous study in Sweden with a large construction worker population (Toren et al. 2007) showed an increase in the risk of ischemic heart disease related to exposure to inorganic dusts, gasses, and fumes. In the present study, we wanted to examine the relation of VGDF to specific causes of death in a general population cohort. We also wanted to study the effect of mineral dust exposure separately, to see whether this alone would have a different influence on mortality compared to overall VGDF exposure.

### Methods

#### Study cohort

**Fig. 1:**
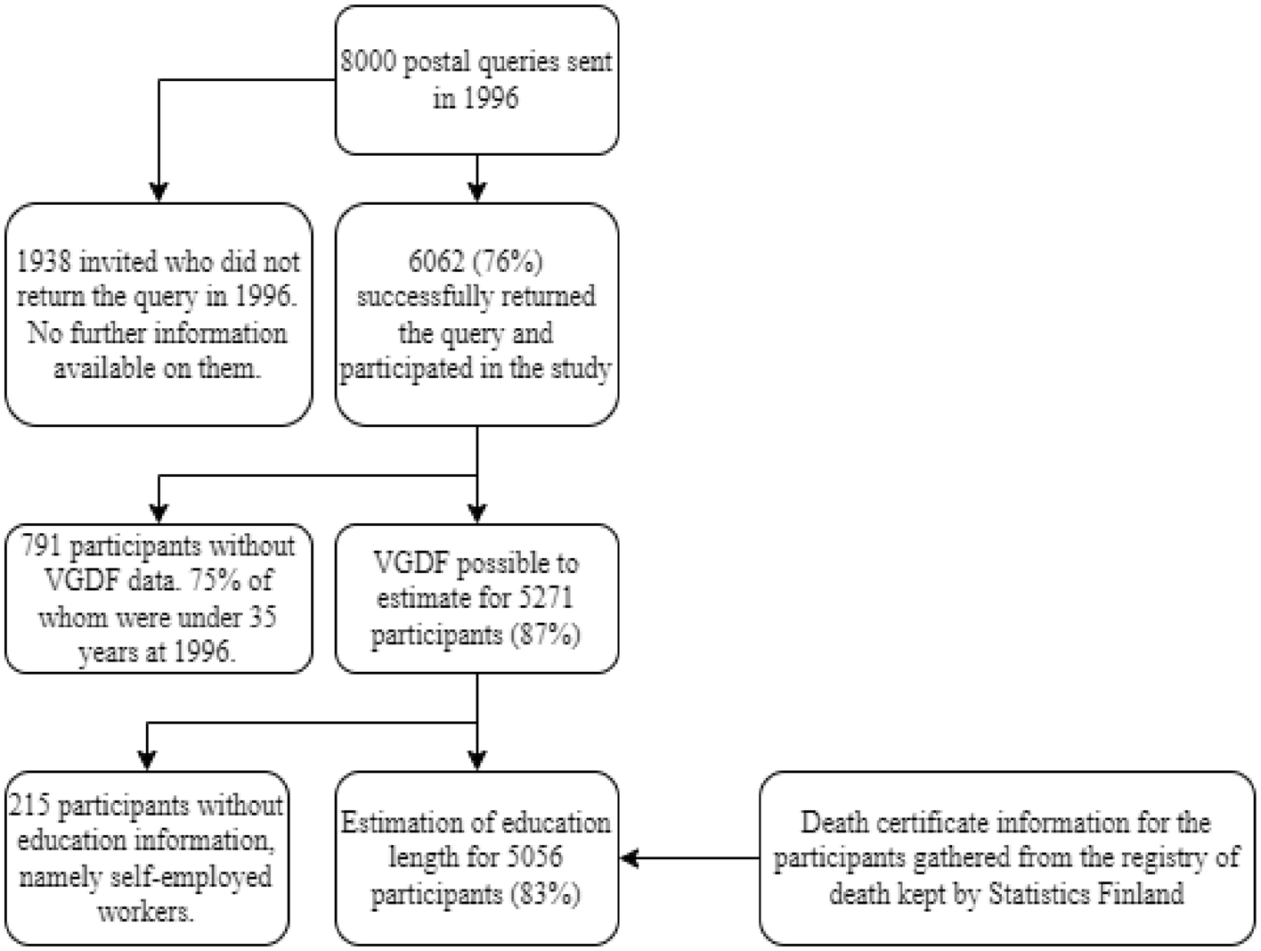
Flow chart of the participants of the study

The study cohort comes from the Helsinki part of the FineEsS study in Finland, Estonia, and Sweden which began in 1996 (Pallasaho et al. 1999). The study population was randomly selected by the Finnish national statistical service (Statistics Finland), aged 20–69 years, in the city of Helsinki in 1996. Postal questionnaires (*n* = 8000) were sent in 1996, 6062 (76%) responded to the questionnaire, among whom 5271 (87%) reported occupation title. The full flow chart can be seen in Fig. 1.

#### Mortality data

Statistics Finland provided the data on cause and date of death, as well as underlying and potentially contributing causes of death coded according to ICD-10, for the full time of follow-up of 24 years, starting from 1.1.1996 and ending 31.12.2019. The definitions for the disease-specific causes of death using both the underlying as well as the contributing causes of death can be seen in Table 1. For the dementia categorization, we used F01, F03, and G30 ICD-10 codes. This categorization of dementia has also been used by the Finnish Institute of Occupational Health in previous studies (Kivimaki et al. 2018, 2019) and exists in the Statistics Finland yearly comparison of mortality in Finland.

**Table 1:** Definitions for specific causes of death according to ICD-10 coding

Cardiovascular mortality	*Cardiovascular-related*	Underlying or contributing causes of death (I00-I99)
	*Coronary artery disease-related*	Underlying or contributing causes of death (I20-I25)
	*Cerebrovascular disease-related*	Underlying or contributing causes of death (I60-I69)
Neurological mortality	*Neurological disease-related*	Underlying or contributing causes of death (G00-G99)
	*Dementia-related*	Underlying or contributing causes of death (F01, F03, G30)
Respiratory mortality	*Respiratory mortality-related*	Underlying or contributing causes of death (J00-J99)
	*Chronic lower airways-related*	Underlying or contributing causes of death (J40-I47)

Coding shown is the ICD-10 (International Classification of diseases ver. 10)

Dementia-related mortality used the specific ICD-10 codes defined by Statistic Finland as being classified dementia

These groups were formed and analyzed separately as one participant could have belonged to several of them.

#### Definitions for smoking status, occupation, and occupational exposure

The main occupation of each participant was obtained by the following question, ‘*What has been your main work/occupation (during your life)?*’. These answers were then converted to both Swedish Socioeconomic Index (SEI) as well as Nordic Classification of Occupations (NYK) coding. We were able to convert these into International Classifications of Occupations 1988 (ISCO88) codes. The corresponding number of cases as well as the information on the conversion can be found in Supplementary Table 1 and Supplementary Fig. 1.

For the occupational exposure, we used a previous VGDF-based Job-exposure Matrix (JEM) [14] on the ISCO88 code. The JEM classified three different types of exposure (biological dust, mineral dust, and gas/fumes). From these, we used mineral dust exposure alone and assessed exposure variable in which gathered the data from all three types. We defined the mineral dust exposure alone and the combined occupational exposure as none, intermediate, and high exposure as seen in Table 2.

**Table 2:** JEM values and the exposure variable

JEM values:	Assessed from the ISCO-88 coding using the two first numbers	Biological dust	0–2 (0 no exposure, 1 intermediate exposure, 2 high exposure)
		Mineral dust	0–2 (0 no exposure, 1 intermediate exposure, 2 high exposure)
		Gasses and fumes	0–2 (0 no exposure, 1 intermediate exposure, 2 high exposure)
Combined Exposure:	Assessed from the JEM values	None:	Value 0 in all three types of exposure
		Intermediate:	Value 1 in any type of exposure but no value 2
		High:	Value 2 in any type of exposure

*JEM* job-exposure matrix; *ISCO-88* International Standard Classification of Occupations ver. 1988

The smoking status used in the analysis was obtained from questions “*Do you smoke? (Smokers also include those who smoke a few cigarettes, or pipe fills a week and those who have stopped smoking during the last 12 months)*” and “*Have you been a smoker but have stopped smoking more than one year ago?*”, and from these, we divided the tobacco smoking status into never-smokers and ever-smokers (including current and ex-smokers). We were also able to obtain age and sex at baseline from the questionnaires.

#### Statistics

Our primary specific mortality model was the Fine–Gray competing-risks regression model (Austin et al. 2016), which is a continuation of the Cox Regression model which compares the cumulative incidences of the disease-specific cause of death between groups, while considering that people with other end-events might also have had a similar hazard but died due to other reasons. If the probability of mortality related to a certain cause is low enough (*p* < 0.2), the sub-hazard results can also be directly interpreted as odds ratio of the event (Austin & Fine 2017). The results therefore are odds for a mortality related to disease-specific cause, comparable to the more widely used Cox model, and expressed as a sub-Hazard ratio (sHR). For confounders, we used age, sex, and tobacco smoking status. Reference group was chosen as having no exposure for the combined exposure groups (no exposure of any VGDF type). For Mineral Dust alone groups, the reference group was no mineral dust exposure. The follow-up time for the study was 24 years.

All statistical analyses were carried out using IBM SPSS Statistics version 27 (IBM Corp, New York, NY, USA), and for the Fine–Gray model, StataCorp. 2021. *Stata Statistical Software: Release 17*. College Station, TX: StataCorp LLC.

## Results

The high exposure group had the highest proportion of current smokers and high current daily cigarette smoking. Table 3 shows the demographics by sex and combined exposure groups.

**Table 3:** Demographic data, smoking habits, use of asthma medication and symptom prevalence in relation to combined occupational exposure and sex

	Sex		Combined exposure		
	Female	Male	No	Intermediate	High
n (%) 5271 (100)	2987 (57)	2284 (43)	2895 (55)	1560 (30)	816 (16)
Clinical characteristics in 1996					
Age (years) Median in 1996 (IQR)	44 (20)	44 (19)	43 (20)	44 (21)	46 (23)
Age (years) range in 1996	20–69	20–69	20–69	20–69	20–69
Sex (female)	2987 (100)	0 (0)	1844 (64)	868 (56)	275 (34)
Tobacco smoking status in 1996					
Never-smoker	1599 (54)	880 (39)	1500 (52)	707 (46)	272 (31)
Ever-smoker	1379 (46)	1396 (61)	1386 (48)	848 (54)	541 (69)
Mortality data during the follow-up					
Age (years) (median) at death	70	72	71	71	70
All-cause mortality in follow-up	427 (14)	511 (22)	426 (15)	284 (18)	228 (28)
Cardiovascular-related mortality	153 (5)	240 (11)	162 (6)	124 (8)	107 (13)
Coronary artery disease-related mortality	84 (3)	146 (6)	83 (3)	76 (5)	71 (9)
Cerebrovascular disease-related mortality	37 (1)	50 (2)	35 (1)	30 (2)	22 (3)
Neurological disease-related mortality	81 (3)	63(3)	70 (2)	43 (3)	31 (4)
Dementia-related mortality	68 (2)	38 (2)	45 (2)	35 (2)	26 (3)
Respiratory-related mortality	82 (3)	132 (6)	84 (3)	72 (5)	58 (7)
Chronic lower airways-related mortality	33 (1)	43 (2)	23 (1)	28 (2)	25 (3)

Data is presented as *n* (%) unless otherwise stated

*COPD* chronic obstructive pulmonary disease: *Shortness of breath* defined as having shortness of breath when walking on a flat ground with own age group*; IQR* Inter-quartile range; *Cig.* Cigarette

The high exposure group was the oldest group as well as had the highest percent of males. They had higher all-cause mortality as well as higher mortality related to all cardiovascular diseases, all neurological diseases, and all respiratory diseases. The high exposure group also had the highest mortality related to just coronary artery diseases (I20–I25), cerebrovascular diseases (I60–I69), and chronic lower airways diseases (J40–J47). They also had highest dementia (F01, F03, and G30)-related mortality.

Males smoked most and were more likely to die and had higher cardiovascular-related mortality as well as respiratory-related mortality.

High exposure and intermediate exposure groups had significant sub-hazard ratios of mortality related to all cardiovascular and coronary artery-related mortality but not cerebrovascular-related mortality. The occupational exposure groups were compared with the Fine–Gray regression model as seen in Table 4.

**Table 4:** Competing risks regression models by disease for mortality in relation to combined exposure

	*n*	*n* events (%)	sHR	95%	CI	*n* events (%)	sHR	95%	CI
	Cardiovascular diseases								
	All cardiovascular-related mortality (I00–I90)					Coronary artery-related (I20–I25)			
No Exposure	2895	162(6)	1	Reference		83 (3)	1	Reference	
Intermediate Exposure	1560	124(8)	**1.30**	**1.02**	**1.64**	76 (5)	**1.55**	**1.13**	**2.12**
High Exposure	816	107(13)	**1.67**	**1.29**	**2.15**	71 (9)	**2.14**	**1.53**	**2.97**
						Cerebrovascular-related (I60–I69)			
No Exposure	2895					35 (1)	1	Reference	
Intermediate Exposure	1560					30 (2)	1.44	0.88	2.36
High Exposure	816					22 (3)	1.53	0.89	2.62
	Neurological and dementia diseases								
	All neurological disease-related mortality (G00-G90)					Dementia-related (F01, F03, G30)			
No Exposure	2895	70(2)	1	Reference		45(2)	1	Reference	
Intermediate Exposure	1560	43(3)	1.08	0.73	1.58	35(2)	1.38	0.89	2.16
High Exposure	816	31(4)	1.24	0.80	1.92	26(3)	**1.70**	**1.04**	**2.76**
	Respiratory diseases								
	All respiratory-related mortality (C00-D90)					Chronic lower airways-related mortality (J40-J47)			
No Exposure	2895	84(3)	1	Reference		23(1)	1	Reference	
Intermediate Exposure	1560	72(5)	**1.43**	**1.04**	**1.97**	28(2)	**2.09**	**1.20**	**3.62**
High Exposure	816	58(7)	**1.72**	**1.22**	**2.41**	25(3)	**2.65**	**1.52**	**4.62**

Statistically significant results are bolded

All models are adjusted for age, sex, and tobacco smoking status

*CI* Confidence interval; *sHR* sub-Hazard ratio

High exposure group had significantly more dementia-related mortality compared to those unexposed, but not in relation all neurological cause mortality. High and intermediate groups had significant respiratory as well chronic lower respiratory disease-related mortalities. If the analyses were stratified by gender, then the results were significant for men only. If no exposure group was formed by no exposure to any of the VDGF (no vapor, dust, gasses, and no fumes exposure), then coronary artery mortality risk for men was 2.17 times higher in the high exposure group. Respectively, the mortality risk for dementia was 4.04 times higher for men in the high exposure group.

The results are like the model with all exposures combined, although the intermediate exposure groups did not have significant respiratory-related sub-hazard of mortality. We were also able to produce a model for mineral dust exposure specifically as shown in Table 5.

**Table 5:** Competing risks regression models by disease for mortality for Mineral Dust Exposure only

	*n*	*n* events (%)	sHR	95%	CI	*n* events (%)	sHR	95%	CI
	Cardiovascular diseases								
	All cardiovascular-related mortality (I00-I90)					Coronary artery-related (I20-I25)			
No Exposure	3693	209 (6)	1	Reference		107 (3)	1	Reference	
Intermediate Exposure	762	77 (10)	1.07	0.81	1.42	52 (7)	**1.43**	**1.01**	**2.03**
High Exposure	816	107 (13)	**1.53**	**1.20**	**1.96**	71 (9)	**1.98**	**1.44**	**2.72**
						Cerebrovascular-related (I60-I69)			
No Exposure	3693					52 (1)	1	Reference	
Intermediate Exposure	762					13 (2)	0.73	0.40	1.35
High Exposure	816					22 (3)	1.20	0.73	1.99
	Neurological and dementia diseases								
	All neurological disease-related mortality (G00-G90)					Dementia-related (F01, F03, G30)			
No Exposure	3693	87 (2)	1	Reference		59 (2)	1	Reference	
Intermediate Exposure	762	26 (3)	1.09	0.59	1.72	21 (3)	1.43	0.85	2.38
High Exposure	816	31 (4)	1.23	0.80	1.89	26 (3)	**1.63**	**1.02**	**2.60**
	Respiratory diseases								
	All respiratory-related mortality (C00-D90)					Chronic lower airways-related mortality (J40-J47)			
No Exposure	3693	111 (3)	1	Reference		35 (1)	1	Reference	
Intermediate Exposure	762	45 (6)	1.18	0.81	1.69	16 (2)	1.48	0.81	2.71
High Exposure	816	58 (7)	**1.55**	**1.12**	**2.14**	25 (3)	**2.12**	**1.29**	**3.48**

Statistically significant results are bolded

All models are adjusted for age, sex, and tobacco smoking status

*CI* Confidence interval; *sHR* sub-Hazard ratio

The high exposure groups had significant sub-hazard mortality related to cardiovascular disease sHR 1.5 (1.2–1.96), coronary artery diseases 2.0 (1.4–2.7), dementia diseases 1.6 (1.0–2.6) and respiratory diseases 1.6 (1.1–2.1) when compared to the no exposure group. Intermediate exposure to mineral dust had only significant sHR 1.4 (1.0–2.0) for coronary artery disease-related mortality.

## Discussion

We compared the sub-hazards of mortality between different levels of occupational VGDF exposure in a large general population cohort during 24-year follow-up time. Our results show higher hazards for disease-specific causes, such as coronary artery disease, dementia as well as chronic lower airway diseases. Of note is the higher sub-hazard mortality in dementia-related causes, which has not been previously shown in an VGDF occupational setting. The long 24-year follow-up in a general population sample of 6062 study participants with combination of VGDF exposure data increases the value of our results.

Previous research on airborne exposure has mostly concentrated on large epidemiological studies on environmental exposure but rarely on occupational exposure. This has likely been due to the relatively easy and reliable access to measures of environmental pollution from automated measuring stations which have been in use for decades. At the same time, information on occupational exposure is usually more difficult to obtain.

Disease-specific causes of death in relation to occupational exposure have been previously studied for ischemic heart disease and cerebrovascular disease (Persson et al. 2007; Toren et al. 2007) and our results are in concordance with that although their studies were concerning an occupational cohort, not a random population as the present material. Also, increased mortality for chronic lower airway diseases in association with occupational environmental exposure has been reported (Bergdahl et al. 2004; Toren & Jarvholm 2014). In an earlier study in the present cohort (Jalasto et al. 2022), we found higher mortality, especially in those with both asthma and COPD, with higher occupational exposure.

As far as we know, there are no follow-up studies on mortality in large general population cohorts which have also included neurological diseases. We utilized the FinEsS study population sample set that was originally designed for respiratory symptom and disease prevalence studies. Some studies have also approximated disease-specific mortality in relation to occupational exposure (Jarvholm et al. 2013; Nurminen and Karjalainen 2001), but these have high-lighted cardiovascular and respiratory causes rather than neurological causes. Some occupational exposure studies have found associations between mortality to different dementia types and occupational exposure to aluminum dust (Peters et al. 2013) or to aluminum and solvents (Graves et al. 1998) or metals, chlorinated solvents, or extremely low-frequency magnetic fields (Koeman et al. 2015). One study found increased incidence of Alzheimer’s disease and dementia with occupational pesticide-exposure (Hayden et al. 2010) and another one association of alumina and bauxite dust exposure and cerebrovascular deaths (Friesen et al. 2009) and a third one suggestive association of arsenic exposure and cerebrovascular deaths (Marsh et al. 2009).

Long-term exposure to particulate matter (PM) in an environmental situation has been previously reported (Wolf et al. 2015) to increase the incidence of coronary events in several European cohorts. In that study, PM at the size 2.5 potassium (K), PM 10 potassium (K), PM 2.5 Iron (Fe), and PM 10 Silicon (Si) had the strongest association with coronary events. A previous study in Japan has also reported increased incidence of acute myocardial infarction, especially non-ST elevated myocardial infarction, associated with environmental exposure (Kojima et al. 2017). In their study, a short-term exposure to dust storm (which contained especially PM2.5, photochemical oxidants, nitrogen dioxide, sulfur dioxide) was found to be associated with increased risk for acute myocardial infarct on the following day. The effect of fine PM to cardiovascular mortality, particularly coronary artery disease mortality, has been previously seen in several studies (Chen and Hoek 2020). Similar findings of cardiovascular mortality can be seen in our study, both by the combined exposure and mineral dust alone exposure groups. The suggested mechanism behind the increased risk for cardiovascular events by PM exposure could be induced oxidative stress and inflammation both in the lungs and systemically and thus promoting atherosclerosis (Wolf et al. 2015). As the exposure in the present study was based on self-reported professions instead of measured concentrations, specific components of mineral dust exposure could not be distinguished.

Neurological mortality generally is less studied and only one study has found significant risk for dementia-related mortality in association with occupational exposure in women (Koeman et al. 2015) though this was specifically for non-vascular dementia. One review suggested that air pollution as a neurotoxic substance would be related to oxidative stress and neuro-inflammation (Costa et al. 2014). A recent review has suggested several methods through which small particle exposure could influence central nervous system (CNS) (Heusinkveld et al. 2016). They proposed the delivery of PM to CNS through olfactory route and systematic route. These have been confirmed in rodent experiments, however, it remains to be determined, how well this could be applied to human subjects. If transport by arteries, the materials would need to cross two barriers, one to enter the blood stream and the other to pass the blood–brain barrier. This would depend on the particles to be either lipid-soluble or having specific transportation methods through the endothelium. However, it is possible that PM promoting inflammation and atherosclerosis may also contribute to neurologic mortality.

Epidemiological studies show that risk for stroke hospitalization or for stroke mortality increases when environmental air levels of PM2.5, PM10, carbon monoxide (CO), sulfur dioxide (SO_2_), and nitric dioxide (NO_2_) level increase (Hahad et al. 2020). Their effect is minor without certain clinical effect at an individual level, but their effect on mortality has been found in large population studies.

Some studies have found an association of PM 2.5 level of fine particles and dementia or Alzheimer’s disease (Costa et al. 2014). Discussion has been whether the neurological effect would be an independent cause or whether air pollution would have a negative effect on cardiovascular health which would then also accelerate cognitive decline and the onset of dementia.

While the exact mechanism of PM to neurological degeneration is not known, our result regarding dementia-related mortality is in line with previous animal studies and as such represents a novel finding in which occupational exposure might have long-term effects also in human brain health. Though we have adjusted the analysis for age, sex, and tobacco smoking status, there might be other confounding factors such as pre-existing cardiovascular disease, particularly atherosclerosis which could have neurodegenerative effect through cerebral small vessel disease contributing to vascular dementia.

The respiratory disease-specific mortality risk in relation to occupational exposure has been shown in previous studies from Sweden (Bergdahl et al. 2004; Toren & Jarvholm 2014), and occupational exposure has been shown to increase the burden of non-malignant lower airway diseases (Baur et al. 2012; Blanc et al. 2019; Paulin et al. 2015). The link between respiratory disease and specifically chronic lower airway diseases and PM exposure is also well-accepted in the large consortiums for asthma and COPD in Global Initiative for Asthma (GINA) Guideline (GINA 2023) and Global Initiative for COPD report (GOLD 2023), respectively. Both organizations also see occupational exposure as an important factor both in the induction of the diseases as well as worsening an existing disease.

The effect of occupational exposure to disease-specific causes of death is notable for cardiovascular and respiratory diseases and has been seen in previous studies. The new finding of dementia-related mortality being affected by occupational exposure is interesting as it has, as far as we know, not been published before though it has been postulated to exist for PM exposure.

### Strengths and limitations

Our study data come from a large well-characterized population-based FinEsS cohort (Pallasaho et al. 1999) with a good responder percentage, originally 6002 answers out of 8000 sent. This alongside the long follow-up time of 24 years allowed us to do reliable statistical analysis with regression analysis and adjust for age, sex, and smoking status. We did not limit the ages of the responders in this follow-up, even though those who were under the age of 40 at the start of the study in 1996 would have been unlikely to develop a dementia causing disease during the follow-up time. Previous non-responder studies with similar postal questionnaire used in Sweden suggested that the effect of non-responders would be non-existent or minimal with regard to airway diseases and symptoms though both showed that young age, male sex, and smoking were significant causes of being a non-responder (Raisanen et al. 2020; Ronmark et al. 2009). The level of diagnostic in Finland is good and follows international guidelines. The Nordic welfare system reduces the socio-economic differences especially regarding the availability of healthcare. In Finland, healthcare professionals usually have the patient information, whether originating from private or public sector, available for them if the patients themselves allow it. This applies to those deceased as well, where the attending physician has the previous diagnostic and patient summaries available to them. Death certificate is mandatory for each deceased and can either be done by the attending physician based on patient information or in unsure cases by professional autopsy report. All certificates are checked by a forensic medical specialist in Finnish Institute for Health and welfare (THL) before processing and kept in a registry by Statistics Finland. This also applies to those who passed away outside of Finland as well, although information passing to the registry is dependent on the next of kin and sometimes on the practices of the country where the deceased lived.

The assessment of diagnosis of various diseases has been based on guidelines produced by their respective fields. These guidelines follow the current up-to-date scientific standards of the respective fields and are updated regularly. Depending on the disease, the first assessment and diagnosis is made by a general practitioner with access to consultation as well as medical imaging and various diagnostic methods from the different fields of specialties. In some cases, the patient is sent to a general hospital for outpatient assessment and diagnosis and to start treatments.

In Finland, each citizen has a personal social security number which can be used to identify the person and link them to different registries. These registries are rigorously maintained and by these identifying numbers medical information can be followed through registries for years.

Our study does not consider the difference between other environmental exposures than occupational exposure between the subjects, and it is possible that it might have also influenced the results. Though the cohort itself is formed from participants who lived in the Helsinki Metropolitan area, different locations in the area may have had slightly different types of environmental exposures especially those alongside heavily trafficked roads and in the city center of Helsinki. One feature that could speak against this is the lower segregation of different socio-economic levels in Finland for the most parts of twentieth and twenty-first century, as both rental flats as well town houses would be mostly in the same areas near each other. Finland also has had one of the best air qualities in the world especially starting from the latter part of the twentieth century.

We were unable to adjust for pack-years of smoking as the data for pack-years as smoking years was not available. Due to only few cases of dementia-related mortality in the highest education group, we did not have the statistical power to perform analyses for education. We were also unable to adjust other risk factors for Alzheimer’s disease including hypertension, type 2 diabetes, obesity, dyslipidemia, as well as stress, depression, and inadequate sleep (Silva et al. 2019). It is also possible that shift work, common in manual labor and as such those with more exposure, could have affected all the mortality computations as shift workers have been seen to have more diabetes as well as myocardial infarctions in a previous study (Karlsson et al. 2005).

### Conclusion

Based on this large random population cohort with a 24-year follow-up, we found that long-term occupational exposure to dusts, gasses, and fumes or mineral dusts alone have increased hazard of mortality for cardiovascular, neurologic, and respiratory disease mortality. This emphasizes the need for minimizing occupational long-term respiratory exposure to dusts, gasses, and fumes.

### Supplementary Information

Below is the link to the electronic supplementary material.

Supplementary file1 (PDF 641 KB)

## Data Availability

The datasets generated during and/or analyzed during the current study are not available due to legal reasons concerning the nature of the data.
