# Supplementary material for: Occupational exposure to vapors, gasses, dusts, and fumes in relation to causes of death during 24 years in Helsinki, Finland
Source: Int Arch Occup Environ Health. 2023 Dec 19;97(2):145–54. doi: 10.1007/s00420-023-02031-1 (PMC10876715; doi:10.1007/s00420-023-02031-1)
Supplement: Supplementary file 1 — Supplementary file1 (PDF 641 KB) [file 420_2023_2031_MOESM1_ESM.pdf]

Supplementary for Airborne occupational exposure and mortality related to disease specific causes in a longitudinal study in Helsinki, Finland:

Supplementary Table 1 shows the n values for the different types of coding. ISCO88 has a lower number due to occupations that are not classifiable in it such as students and retirees, unemployed and long-term pensioners without information on their main occupation. It also has included links to the corresponding classification information as well information on the ISCO88 transformation form. It should be noted that the NYK coding in this data was done with the Swedish version rather than the Finnish version which slightly differs even though the data originates from Finland.

Supplementary table 1. Occupational coding definitions

SEI (Swedish Socio-economic index)[1]	Two-to-three-digit coding from the original occupational title. Done previously by researchers in the 1990s based on the main occupation title	*n = 6062*
NYK (Nordic Classification of Occupations)[2]	Four-digit coding from the original occupational title. Done previously by researchers in the 1990s based on the main occupation title	*n = 6062*
ISCO-88 (International Standard Classification of Occupations, 1988 version)	Transformation done in 2021, using the original SEI and NYK codings[3]	*n = 5271*

[1] The Swedish Socio-Economic classification: Rationale and Fields of application
(_) (Last accessed 17.01.2022)
[2]https://www.scb.se/hitta-statistik/aldre-statistik/innehall/sveriges-officiella-statistik-sos/folk-och-bostadsrakningarna/1965-1990/1985/ (Last accessed 17.01.2022)
[3]Transformation form by Erik Bihagen: (https://www.camsis.stir.ac.uk/occunits/distribution.html#Sweden) (Last accessed 17.01.2022)

Figure 1 JEM assignment and exposure variable compilation.

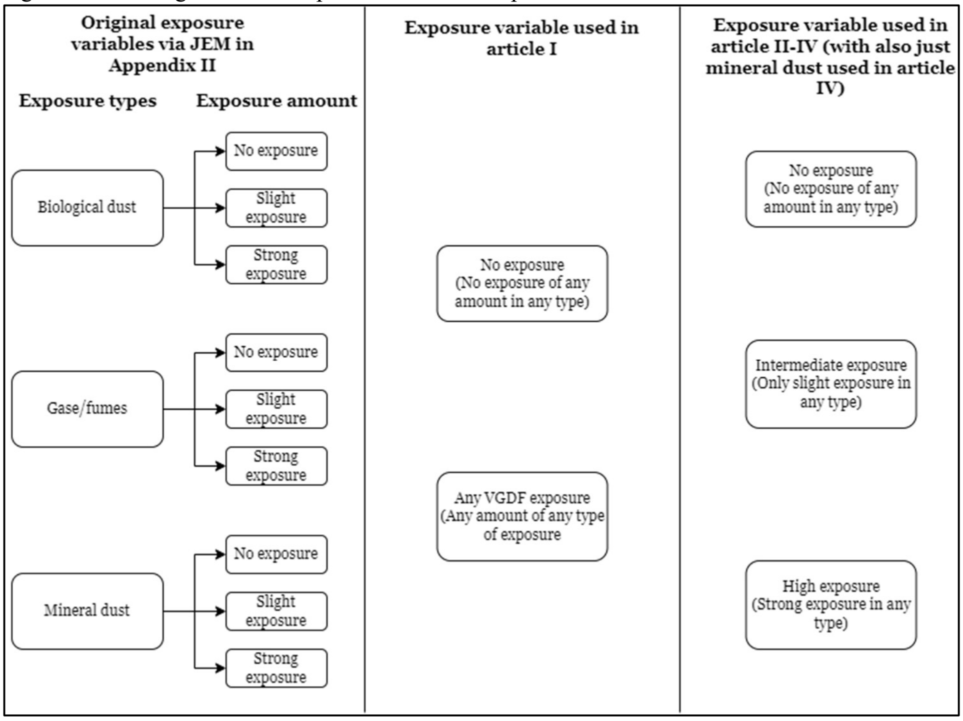

Supplementary table 2. Dementia related mortality code break-up

	Sex		Combined exposure		
	**Female**	**Male**	**No**	**Intermediate**	**High**
n (%) 5271 (100)	2987 (57)	2284 (43)	2895 (55)	1560 (30)	816 (16)
Dementia related mortality (F01, F03, G30)	68 (2)	38 (2)	45 (2)	35 (2)	26 (3)
Alzheimer's disease related mortality (G30)	57 (2)	27 (1)	37 (1)	27 (2)	20 (3)
Vascular dementia related mortality (F01)	9 (0)	7 (1)	6 (0)	6 (0)	4 (1)
Unspecified dementia related mortality (F03)	2 (0)	4 (0)	2 (0)	2 (0)	2 (0)

There were no participants with both G30 and F01 or F03 in their death certificates (including contributing causes).

Supplementary Table 3. Competing risks regression models by disease for mortality in relation to combined exposure

Alzheimer's and all dementia related mortality

		Alzheimer's disease related (G30)				Dementia related (F01, F03, G30)			
No Exposure	2895	37(1)	1	Reference		45(2)	1	Reference	
Intermediate Exposure	1560	27(2)	1.30	0.79	2.13	35(2)	1.38	0.89	2.16
High Exposure	816	20(3)	1.62	0.94	2.79	26(3)	**1.70**	**1.04**	**2.76**

Statistically significant results are bolded
All models are adjusted for age, sex and tobacco smoking status
CI Confidence interval; *sHR* sub-Hazard ratio

Figure 1. Kaplan-Meier survival estimates for those 50 years old and over in relation to combined exposure

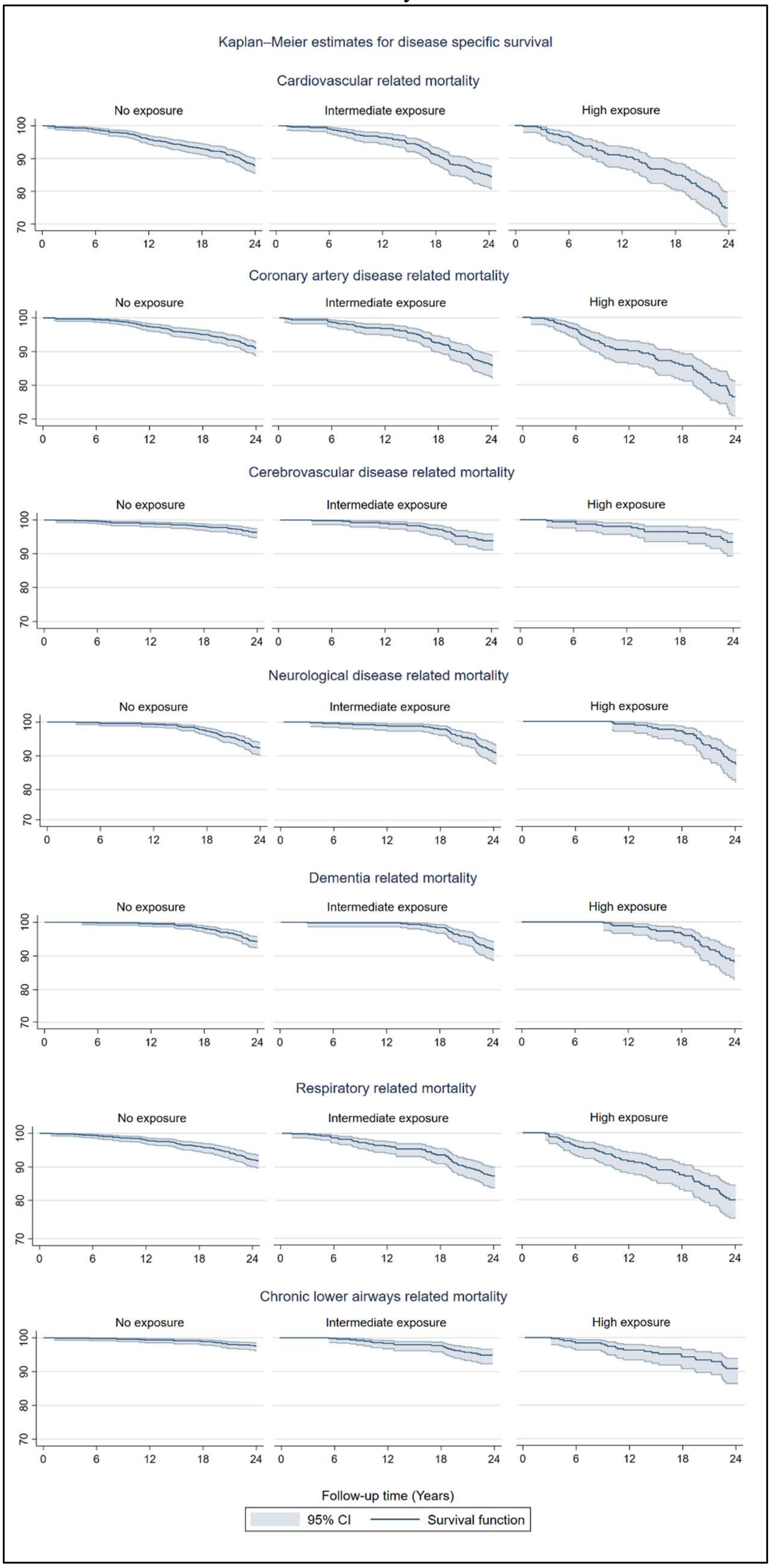

Similarly, to the Competing risks regression analysis, the high exposure group shows the lowest survival estimate in all causes of death related to specific disease groups. Supplementary Figure 1 shows all the disease specific Kaplan-Meier survival estimates for those 50 years old and over. Cardiovascular related mortality began earlier in the follow-up then cerebrovascular or neurological disease related mortality.
